# Selections

**Published:** 1816-02

**Authors:** 


### PART III.

### SELECTIONS.

### FRENCH DICTIONARY OF MEDICAL SCIENCES.

( Continued from p. 80. )

2. *Asphyxia from Suffocation.* This may take place slowly or instantaneously; and the effects differ. A foreign body may get into the trachea, but not completely obstruct the passage of air: respiration goes on imperfectly. Cough, convulsion, turgescence, and lividity of countenance, ensue. After death, the lungs are found gorged with blood and froth ; heart greatly distended ; its contractility promptly annihilated. But, if the aerial tube be completely obstructed, sensation and movement are soon lost: the face reddens ; the eyes become fixed and prominent ; but the heart long preserves its faculty of contracting under stimulants; the lungs are less gorged, and without frothy matter. The hope of resuscitation is here much greater. In this kind of asphyxia, the treatment should first be directed to the removal of the cause. With this, the symptoms will commonly disappear : otherwise, irritants, local bleeding, and vomits, may be advantageously employed. With respect to the adoption of the latter remedies, under some circumstances prejudicial, the experience of the practitioner must decide.

3. *Asphyxia from drowning.* Much dispute has arisen respecting the cause of death in drowned animals. Goodwyn proved by experiment, that, in these cases, a certain quantity of water enters into the lungs before death ; but not sufficient to determine that event. It was reserved for French genius to perfect the incomplete experiments of the British philosopher, and solve every difficulty. M. Berger repeated the experiments of Goodwyn, and instituted others. By examining the gas expired by drowning animals, he discovered, that they only became asphyxiated when the air contained in the lungs was so vitiated, as no longer to affect the coloration of the blood, and that then only they discharged it. These are the phænomena exhibited by a drowning animal : pulse feeble and frequent ; oppression of the chest ; agitation, and an attempt to gain the surface of the liquid ; escape of air from the lungs ; increased anxiety and depression of pulse ; struggling more violent, and discharge of air more copious ; efforts to respire ; introduction of water into the mouth and trachea ; skin, particularly face and lips, becoming blue ; pulse intermitting ; sphincters relaxed ; loss of sense and motion. These phænomena vary, according to circumstances, in the rapidity of succession. Asphyxia takes place in about three minutes when the animal is kept immersed. Dissection shews the right cavities of the heart and their large vessels distended with black blood ; the left much less so, sometimes quite empty ; the irritability of the heart (soon after death) not quite extinct, particularly in the right cavities, the contractions of which may be excited by galvanism for a long time ; much frothy fluid in the lungs ; the pulmonary veins and arteries filled with black blood : surface of the brain of a colour more obscure than common, but no congestion in its vessels, no extravasation in its substance. The directions respecting the treatment are copious and vary little from those delivered in English works. Shaking the body and suspending it by the heels, are deservedly reprobated. It is to be laid on a low bed somewhat raised towards the head ; the clothes immediately removed. Careful examination should be made, lest any wound exist, capable itself of destroying life ; and thus necessarily foiling the efforts at resuscitation. Heat should be applied to the epigastrium, feet, and successively to other parts: frictions with the hand or a flannel, at first dry, then moistened with camphorated alcohol, ammonia, vinegar; irritation of the nasal membrane by ammonia, vapours of sulphur, or oxymuriatic acid gas; titillation of the lips and nostrils with a feather; cautious introduction of stimulants into the stomach by means of an elastic gum catheter passed into the œsophagus, to prevent the ill consequences of their passage into the larynx; irritating injections of marine salt in vinegar and water, or decoctions of tobacco;* inflation of the lungs by the mouth, bellows, or a tube passed into the larynx, are the remedies principally recommended. When appearances of returning life arise, a light cordial should be administered. The injection of tobacco-smoke per anum may be hurtful by distending the large intestines and thrusting up the diaphragm towards the chest. Bronchotomy† offers no particular advantages: bleeding is not indicated in this kind of asphyxia. Vomiting should only be excited when some particular state of the stomach demands it, and there is no fear of cerebral congestion.

4. *Asphyxia from strangulation.* This is an uncertain kind of death. Luxation of the atlas does not necessarily take place. It is a real asphyxia determined by constriction of the trachea; and, in many points, analogous to that consequent on drowning; but complicated with congestion of venous blood in the cerebral vessels, from compression of the jugular veins. Convulsive motions; swelling of the face; projection of the eyes; tumefaction and protrusion of the tongue; erection of the membrum virile; ecchymosis of the scrotum; cessation of pulse; and annihilation of motion, are the phænomena displayed in a man, upon suspension. Some, who have been restored to life, declare that they felt no pain, but a sort of numbness, succeeded by total insensibility. Others have perceived a flash of light, succeeded by utter darkness. On dissection have been observed congestion of the cerebral vessels; sometimes extravasations of blood or serum in the interior of the cranium; the lungs and heart, particularly the left cavities of the latter, gorged with black blood. The body long retains its natural warmth. The means indicated in drowning are, with some modifications, to be employed here. Friction is less necessary; and the cerebral congestion demands commonly local or general bleeding: The constitution and state of the subject must be consulted. In some cases, loss of blood would be detrimental. A case is de-

* This is now justly denounced among us as dangerous practice. The employment of a powerful narcotic in cases of suspended animation, even theoretically considered, is irrational. We have known a rupture-patient destroyed by a *common* dose of tobacco-infusion, thrown into the rectum.

† There is no such operation as *bronchotomy*. We had hoped, long ere this, to have seen a term, inaccurate as useless, for ever banished from surgical writings.

tailed in which copious bleeding and repeated purgatives were successful. Delirium came on, and was succeeded by a kind of idiotism, which gradually subsided.*

Asphyxia from want of respirable air. We have hitherto been considering the asphyxia determined by mechanical violence. We have now to examine those, the causes of which exist in the fluid itself, received into the lungs. These may be called the *gaseous* asphyxiæ. They are of three kinds, as the gas is simply unfit for respiration, irritating, or deleterious. The irrespirable produce asphyxia, because they contain not the principle (oxygen gas) necessary to convert the venous blood into arterial; or because it is in a state of combination too intimate to act upon the blood.

1. *Asphyxia from nitrogen gas.* This gas is one of the causes of the *plomb* or asphyxia of privies. Small animals, plunged into an atmosphere of nitrogen, have been asphyxiated in from three minutes and a half to five and a half; some say, probably erroneously, three quarters of an hour. The event will be more prompt in proportion as the lungs have been previously emptied of common air. The symptoms are, disordered respiration, immediately on immersion in the gas, becoming unusually deep and rapid; progressive debility without lesion of the nervous functions. Restoration to common air speedily and completely dissipates this asphyxia, if it be not quite confirmed. Dissection shews the arterial system filled with black blood: the muscles, according to Hallé, scarcely contracting under the influence of galvanism, are yet very sensible to mechanical agents. Nysten, on the other hand, asserts, that in these cases, galvanic excitation is longest felt. Restoration to air is the principal remedy, and commonly efficacious in slight cases. Stimulants may also be resorted to.

2. *Asphyxia from hydrogen gas.* Philosophers are not agreed as to the time in which animals are asphyxiated by this gas. It may be respired for some time with impunity by man: but in this experiment the hydrogen must not be mixed with oxygen or atmospheric air, lest a dangerous explosion ensue, as happened to Pilatre de Rozier. Fontana asserts, that it cannot be respired without danger, even when there is no air in the lungs. Davy could inhale

---

* Hanging is indeed an uncertain and barbarous mode of destroying life; hence highly objectionable as an instrument of legal vengeance. The mind revolts with horror and disgust from contemplating the dreadful scenes which many of our provincial executions have, within a few years, exhibited. Such occurrences, it may be said, were purely accidental; but what consolation is this to the tortured criminal—what relief to the outraged feelings of his relatives! Ignorance and neglect, on such occasions, constitute no venial offence, but a deep and aggravated crime. Would that it were in our power to attract the attention of the great and philanthrophic of the land to this subject; and awake, in behalf of the poor condemned culprit, the eloquence of a British senate!

it but for half a minute. The symptoms which he felt were, uneasiness in the breast, momentary loss of muscular power, and transient vertigo. The blood, on dissection, appears thick and black: muscular irritability appears to remain longer than in the asphyxia produced by drowning, or by inhalation of nitrogen gas.

3. *Asphyxia from nitrous oxide.* The propriety of arranging this gas, as productive of asphyxia, is questionable; for it acts upon the nervous system, and does not sensibly change the colour of the blood. As the extraordinary and very variable effects of this gas upon the human system are well known to our readers, we may be excused from transcribing a long account of the experiments of a society of amateurs at Thoulouse, much more amusing than instructive. A bird was killed by a second dose of this *celestial fluid* of our air-brained poet-laureat.

4. *Asphyxia from carbonic acid gas.* The effects of this gas have been confounded with those resulting from inhalation of nitrogen and hydrogen. They are commonly observed among brewers, or in cellars above vats during fermentation, in lime-kilns or certain subterraneous cavities, as in the Grotto del Cani near Naples. The results of experiments, instituted to determine the effects of this gas upon respiration, differ. Hallé observed a small animal completely asphyxiated in two minutes. Bichat asserts, that animals may respire it more than twice that time; Varin, that when mingled in the proportion of two or three per cent. with atmospheric air, it is harmless; but that, when mixed with four times its bulk of oxygen gas, it asphyxiates in two minutes. The body, after death, long retains its natural warmth; muscular irritability remains more than twenty-four hours: the blood-vessels, particularly those of the lungs, are gorged with blood of a deeper colour than in asphyxia from nitrogen or hydrogen gas, but less black than when death has been produced by respiration of carburetted hydrogen, or carbonous oxide. The treatment consists of exposure to the open air; dashing cold water on the face and epigastrium; irritating the nostrils, and plying all the other means indicated in suffocation. Nothing should be neglected until general rigidity of the muscles, the only real characteristic sign of death, has taken place.

5. *Asphyxia from air vitiated by respiration.* Here, two of the causes of asphyxia, already examined, are combined—the presence of nitrogen and carbonic acid gas; but to the latter the noxious effects are principally to be ascribed. As illustrative of this species of asphyxia, the dreadful fate of our unfortunate countrymen confined in the Black Hole at Calcutta, is minutely detailed. We need not retrace a tale of horror so deeply engraven on the heart of every Englishman. The symptoms, displayed by animals breathing unrenewed air, are, first, inquietude; accelerated respiration and pulse; afterwards, respiration becoming slower; and stupor, shortly terminated by death. A cat, ten inches long, confined in two hundred and seventy-two cubic inches of air, was quite asphyxiated in eighteen minutes; recovered by inflation of the lungs, on being withdrawn ten minutes after; but died at the end of nine hours in convulsions. Analysis of the air expired by animals in these experiments, affords from one to thirteen per cent. of oxygen, instead of the twenty-two originally contained. The quantity of carbonic acid gas cannot be accurately ascertained: it seems to vary from seven to twelve per cent. Dissection shews much black blood in the right cavities of the heart and whole venous system: the left cavities, the aorta and its branches, contain it in less quantity. For the treatment, we are referred to the preceding paragraphs.

Asphyxia from irritating gases. These are the sulphurous acid, oxymuriatic acid, and ammoniacal gases. Little is known of their action. They provoke cough; and, when respired alone, soon destroy life. The first, according to Hallé, killed small animals in a minute and a quarter; the second, in two minutes and a half; the last, in one minute. To the influence of ammoniacal gas, Dupuytren attributes the affection of the *conjunctiva*, to which nightmen are subject. Mixed in proportion of a tenth part with common air, it is innoxious. The proper remedies are not determined. In addition to exposure in air, the inhalation of air loaded with aqueous vapours seems indicated. Chemistry also offers here means of directly obviating the cause of asphyxia, by the extrication of alkaline or acid vapours, according to the nature of the asphyxiating gas.

Asphyxia from deleterious gases. These act not only by subtracting from the lungs the respirable principle, irritating them, and producing death by the sympathy which exists between them and other organs; but they exert an influence on the whole œconomy, seemingly by absorption. Only respecting three or four of these have any certain notions been acquired.

1. *Asphyxia from nitrous gas.* This gas differs, in properties as in composition, from the nitrous oxide. One cat was exposed by Berger in an atmosphere of pure nitrous gas; another, in equal parts of this and hydrogen: both died, in half a minute, in terrible convulsions. In the first, the lungs were liver-coloured; the heart ceased to contract in fifteen minutes after death. In the second, the lungs were natural; the heart contracted for half an hour. Nysten, by injecting this gas into the venous system, produced effects resembling asphyxia; the symptoms, embarrassed respiration; hard cough; general debility progressively increasing, with moans; pulse small and unresisting; limbs bending beneath the weight of the body, which became cold in a remarkable manner; pulse, after a time, insensible; respiration deep, infrequent, and rattling; the arterial blood, examined at different times, appeared brown or chocolate coloured; it coagulated on exposure to air, without assuming, on the surface, the vermilion hue common to unaltered venous blood, or to arterial blood blackened in asphyxia from drowning. Death happened more or less rapidly, according to the strength of the animal and quantity of gas injected: the lungs were marbled with livid-red, and gorged with blood and frothy mucus. A case is quoted from the Journal de Médecine; in which a man died twenty-seven hours after inhalation of this gas. The symptoms were great debility, heat and dryness in the throat; irritation in the stomach and chest: constriction of the epigastrium; urine scanty; and, after a time, strangury; expectoration of yellowish matter; cough; nausea and vomiting. After seventeen hours, the face became blue; chest embarrassed; rattling; hiccough; pain in the region of the diaphragm; convulsions; slight delirium, and inexpressible agony. The belly swelled remarkably after death; countenance became purple; lips black; blood oozed from the nose and mouth. The body was not examined.

2. *Asphyxia by carbonous oxide and carburetted hydrogen gas.* It is not known to which of these gases the pernicious effects of the vapours of charcoal are attributable. Probably, both contribute. Both are disengaged from burning charcoal, both are more or less deleterious. When mixed with atmospheric air the action of these gases is rarely sudden: violent head-ach, compression about the temples; vertigo; palpitations; noise in the ears; sometimes nausea; difficult respiration, disorder and loss of vision; prostration of strength, and inability to stand, are the symptoms. Both these gases impart a brown tinge to the arterial blood: this tint is deeper with the first, than the second. With respect to the property of colouring the blood, Nysten places carbonic acid gas between the carburetted hydrogen and carbonous oxide. The bodies of those asphyxiated by charcoal-vapour, long preserve their warmth: sometimes, the temperature is præternaturally increased: the countenance is swollen and red; eyes lively; lips vermilion: body tumefied, often marked with violet-coloured spots: the venous system filled with black and fluid blood; arteries empty or almost so: pulmonary and cerebral vessels particularly gorged.—Treatment: Prompt exposure to open air without fear of cold; potions of vinegar diluted with water; injections of cold water; friction with cloths moistened in oxycrat: In bad cases, attended with drowsiness, bleeding in the jugular vein or foot, especially if the face be red, the lips swollen and eyes prominent; vomits, if there be distension of the stomach, and nausea; purgative injections generally preferable; stimulants. Spirituous liquors and a very warm bed are injurious.

3. *Asphyxia from sulphuretted hydrogen gas.* To this gas is principally owing the asphyxia of privies. The symptoms are, peculiar uneasiness; pain of the stomach and joints; obstructed respiration; drowsiness; delirium; convulsions; loss of memory. There is something contagious in this kind of asphyxia. Dupuytren found that in air, containing from $\frac{1}{5}$ to $\frac{1}{800}$ of this gas, birds were irrecoverably asphyxiated in a few seconds; that in the proportion of $\frac{1}{1000}$, it only affected respiration without producing death. Dogs respired with impunity $\frac{1}{400}$; but, mixed in the proportion of one part to two hundred and ninety-nine of atmospheric air, it asphyxiated one of these animals. Another, more strong, only experienced similar effects from air containing $\frac{1}{100}$ of sulphuretted hydrogen. Dissection shews the bronchia and nasal cavities smeared with a viscous and brownish mucus; the blood filling the vessels, black and thick; muscles blackish and utterly deprived of their contractility; soft parts without the natural consistence, readily tearing, and speedily running into putrefaction. Asphyxia is determined, though somewhat more tardily, by injection of this gas into the cellular membrane, intestinal canal or blood-vessels, or by immersion of a considerable part of the body in it. In all these cases, nearly the same morbid appearances are observed.—Treatment: exposure to air; aspersion of cold water; frictions with vinegar. The workmen, exposed to the action of this gas, employ a vomit of oil olive in the earlier stages; giving, afterwards, a little brandy. Dupuytren has successfully employed oxy-muriatic gas to destroy the effects of the sulphuretted hydrogen. Promptitude in its application is necessary. The former of these gases is preferable to ventilation in counteracting the deleterious influence of the sulphuretted hydrogen.

4. *Asphyxia from hydro-sulphuret of ammonia.* This gas is a compound of ammonia and sulphuretted hydrogen: hence, we may appreciate its effects. Both of the latter gases exist in privies. The presence of the ammoniacal aggravates little the deleterious influence of the sulphuretted hydrogen; but renders more difficult the destruction of the latter by oxy-muriatic acid gas. Two parts of this, according to Dupuytren, are required completely to decompose one part of sulphuretted hydrogen; but a much greater quantity is necessary to effect this decomposition, when the sulphuretted hydrogen is combined with ammonia.

The résumé of our author we shall not copy. It presents nothing important; and we have already extended this article beyond the limits we had prescribed. The subject, however, is peculiarly interesting. We shall endeavour to be more concise, for the future. The monographs on asphyxia here enumerated, are those of *Goodwyn*, London, 1788; *Curry*, London, 1799; *Varin*, Paris, 1802: *Graf*, Strasburgh, 1803; *Freteau*, Paris, 1804; *Berger*, Paris, 1805; *Portal*, Paris, 1811. A second edition of Dr. Curry's work has been lately published. The articles, *Asphyxia*, with their relatives in the *Edinburgh*, and *London*, *Medical Dictionaries*, may also be consulted. Cases of asphyxia by charcoal-vapour are recorded by *Larrey*, *Memoires de chirurgie militaire*, tome, 3 Page 13; by *Dr. Babington*, *Medico-Chirurgical Transactions*, vol. 1. Page 83; Cases of idiopathic asphyxia, by *Chevalier*, same work and volume. Page 157.

PART

